# What affects programme engagement for Māori families? A qualitative study of a family‐based, multidisciplinary healthy lifestyle programme for children and adolescents

**DOI:** 10.1111/jpc.15309

**Published:** 2020-12-23

**Authors:** Cervantée EK Wild, Ngauru T Rawiri, Esther J Willing, Paul L Hofman, Yvonne C Anderson

**Affiliations:** ^1^ Liggins Institute University of Auckland Auckland New Zealand; ^2^ Department of Paediatrics: Child and Youth Health, Faculty of Medical and Health Sciences University of Auckland Auckland New Zealand; ^3^ Kōhatu – Centre for Hauora Māori University of Otago Dunedin New Zealand; ^4^ Starship Children's Hospital Auckland District Health Board Auckland New Zealand; ^5^ Tamariki Pakari Child Health and Wellbeing Trust New Plymouth New Zealand; ^6^ Department of Paediatrics Taranaki District Health Board New Plymouth New Zealand

**Keywords:** health equity, intervention, obesity, racism, retention

## Abstract

**Aim:**

It is important that intervention programmes are accessible and acceptable for groups most affected by excess weight. This study aimed to understand the barriers to and facilitators of engagement for Māori in a community‐based, assessment‐and‐intervention healthy lifestyle programme (Whānau Pakari).

**Methods:**

Sixty‐four in‐depth, home‐based interviews were conducted with past service users. Half of these were with families with Māori children and half with non‐Māori families. The interviews were thematically analysed with peer debriefing for validity.

**Results:**

Māori families experienced barriers due to racism throughout the health system and society, which then affected their ability to engage with the programme. Key barriers included the institutionalised racism evident through substantial structural barriers and socio‐economic challenges, the experience of interpersonal racism and its cumulative impact with weight stigma, and internalised racism and beliefs of biological determinism. Responses to these barriers were distrust of health services, followed by renewed engagement or complete disengagement. Participants identified culturally appropriate care as that which was compassionate, respectful, and focused on relationship building.

**Conclusions:**

While Whānau Pakari is considered appropriate due to the approach of the delivery team, this is insufficient to retain some Māori families who face increased socio‐economic and structural barriers. Past instances of weight stigma and racism have enduring effects when re‐engaging with future health services, and inequities are likely to persist until these issues are addressed within the health system and wider society.

## What is already known on this topic

1. Indigenous peoples face increased barriers to accessing health care due to the ongoing effects of colonisation.
2. While Indigenous groups and other ethnic minority groups tend to have lower retention rates in healthy lifestyle programmes addressing childhood obesity, previous research has not examined the underlying factors or simply cited ‘cultural inappropriateness’ of the programme without elaboration.
3. Racism is a known determinant of health and driver of inequities for Indigenous peoples.

## What this paper adds

1. Engagement in multidisciplinary healthy lifestyle programmes is affected by experiences of institutionalised, interpersonal and internalised racism for Māori families.
2. Racism which occurs elsewhere in the health system or in wider society may have ongoing effects on subsequent engagement with other health services, and until these issues are addressed, inequities in service engagement between Indigenous and non‐Indigenous groups are likely to persist.
3. Compassionate, respectful, appropriate and culturally safe care focusing on positive relationship‐building can help mitigate some of the impact of racism on engagement in prevailing health care services.

While high prevalence rates of obesity are an issue for all groups, inequities persist globally in both prevalence and access to health care, across ethnicity, gender, and socio‐economic status.^1^ In Aotearoa New Zealand (NZ), obesity rates are 1.6 times higher in Māori (the Indigenous people of NZ) children and 4.7 times higher in Pacific children compared with the overall population of children aged 2–14 years.^2^ [Correction added on 23 April 2021, after first online publication: The ratio of obesity rates in Pacific children compared with the overall population has been changed from 3 to 4.7 times.] Children living in the most socio‐economically deprived areas are also twice as likely to experience obesity than those living in the least deprived areas.^2^ These findings are consistent with international inequities between Indigenous and non‐Indigenous populations.^1^

Multidisciplinary intervention programmes remain recommended best practice for addressing childhood obesity,^3^ and retention is key to positive health outcomes.^4^, ^5^ However, previous research also demonstrates inequities in attendance between different ethnic groups – a systematic review of ‘barriers’ and ‘facilitators’ to participation within paediatric weight management programmes showed higher dropout rates among Black participants and those on a low family income than White participants in the USA.^6^ However, few studies have explored the underlying reasons for differences in attendance among Indigenous and other marginalised ethnic groups, or have cited ‘cultural inappropriateness’ of the programme as the reason for disengagement, without elaboration.^4^, ^7^

Indigenous peoples face increased barriers to accessing health care.^8^ Racism is a known determinant of health and driver of inequities for Indigenous peoples, and is increasingly acknowledged as shaping health care interactions and outcomes.^9^, ^10^ *Institutionalised racism*, as an ongoing effect of the colonisation of Indigenous peoples world‐wide, is reflected in differential access to social, political and economic resources and poorer health and social outcomes. It can operate without identifiable individual perpetrators,^11^, ^12^ and may manifest in poorer material conditions. *Interpersonal racism* refers to personally‐mediated discrimination and may appear in the health system in a more covert and passive form, manifesting as *implicit bias*, a belief or association about a social group that may be automatic.^13^ Lastly, *internalised racism* refers to the acceptance and internalisation of racial assumptions and stereotypes by groups themselves in society.^9^, ^10^, ^12^, ^13^ In NZ and other countries with similar colonial histories, these varying forms of racism are entrenched in the context of colonisation and the historical and ongoing processes of dispossession and marginalisation.^14^ This has resulted in ingrained structural inequities within the health system that give rise to inequitable outcomes across a range of areas, such as access to primary health care.

Māori report a higher prevalence of racial discrimination and are also more likely to experience multiple forms of discrimination compared with NZ Europeans.^15^ Racism is known to be associated with both poorer health outcomes and reduced access to health care and resources.^16^ Under the Treaty of Waitangi, and reinforced by commitments to international conventions such as the United Nations Declaration on the Rights of Indigenous Peoples and the United Nations Convention on the Rights of the Child, health professionals and researchers in NZ have an obligation to address health inequities for Māori.^17^ One of the guiding principles of the NZ Health Strategy is equitable access to health services,^18^ to improve health outcomes for those most affected by conditions such as obesity.

Whānau Pakari is a family‐based intervention programme for children/adolescents with weight issues, with a multidisciplinary team including a paediatrician, physical activity specialist, dietitian, healthy lifestyles coordinator, and a clinical psychologist.^19^ It aims to be non‐stigmatising, with a focus on healthy lifestyle change rather than weight or obesity. Programme conception and consultation involved key Māori community stakeholders in the region and is ongoing.^19^ The programme has replaced the conventional hospital‐based model of care within the prevailing health care model in Taranaki, NZ, in order to provide more accessible, appropriate health care. The results of a randomised clinical trial embedded in the service found that participants achieved a modest reduction in body mass index standard deviation score, as well as achieving positive changes in health‐related quality of life and cardiovascular fitness.^5^ Greater reductions in body mass index standard deviation score were achieved if participants attended ≥70% of programme sessions.^5^, ^20^ While service recruitment included approximately equal numbers of Māori and NZ European participants, Māori were less likely to attend the recommended optimal number of sessions.^5^ A previous paper describes the experiences of Whānau Pakari participants with varying levels of attendance.

In NZ, health research should be responsive to the needs and diversity of Māori.^17^ The approach of this research team was based on Kaupapa Māori theoretical principles.^17^, ^21^, ^22^ It was aligned with social and structural determinants of health approach and developed with the aim of contributing towards the elimination of health inequities for Māori, resisting persistent power imbalances and the continued use of cultural deficit theory to explain inequities between Māori and non‐Māori, which attributes poor Māori health to Māori ‘culture’ or something inherent to Māori as a group.^17^, ^22^ The aim of this paper was to explore the experiences of Māori families referred to Whānau Pakari. Given the suggested relevance of ‘cultural appropriateness’ by previous studies,^4^, ^7^ we also sought to determine whether Whānau Pakari was perceived to be appropriate for Māori.

## Methods

The method for conducting the interviews has been described previously.^21^ Briefly, authors 1 and 2 undertook 64 in‐depth interviews in the home with past participants of Whānau Pakari of varying levels of engagement, with equal numbers of interviews with families with Māori and non‐Māori children (Table 1). Interview participants were mainly parents, grandparents and/or caregivers of children involved in Whānau Pakari (five children/adolescents themselves participated in the interviews) and were from a range of socio‐economic backgrounds (deciles 1–10 of the 2013 NZ Index of Deprivation).^23^

**Table 1:** Participant demographics

Interview participants, *n*	
Female participant, *n*	76†
Ethnicity, %‡	65
Māori	32
NZ European	75
Asian	7
Other European	5
Level of engagement, *n*	
Attended ≥70% of programme sessions§	18
Māori	8
Non‐Māori	10
Attended <30% of programme sessions¶	19
Māori	10
Non‐Māori	9
Had one assessment, then discontinued with the programme††	7
Māori	4
Non‐Māori	3
Referred, but chose not to engage‡‡	20
Māori	10
Non‐Māori	10

^†^ Sixty‐four interviews total, 11 interviews involved 2+ family members. Half of the interviews were with families with Māori children. Five interviews included a child/adolescent participant in addition to their parent/caregiver. Maximum total of 74 potential interviews for funding and resource reasons. A total of 136 families approached, of which 53 were uncontactable, 7 were living out of the region, and 12 declined (see COREQ checklist for reasons).

^‡^ Total ethnicity output (more than one ethnicity selected). Twelve participants identified with more than one ethnicity.

^§^ Twenty‐four families invited total.

^¶^ Forty‐two families invited total.

^††^ Fifteen families invited total.

^‡‡^ Fifty‐five families invited total.

Interviews centred on families' experiences of engaging in Whānau Pakari and the wider health system. Interview recordings were independently transcribed, and participants were offered their transcripts to check for accuracy and acceptability. Participants were offered a koha (gift, contribution) as a sign of reciprocity for their time and for sharing their experiences.

The transcripts were coded and analysed in MAXQDA software by author 1 using thematic analysis, identifying common patterns across participants as well as any potential differences between groups. All authors collaborated to finalise the themes and develop the framework, with agreed respectful parameters allowing the authors to debate, challenge and refine interpretations of the data. The researchers agreed to apply the ‘Give‐Way’ rule if there was disagreement over the interpretation of the data concerning Māori participants, and the final decision involving cultural interpretation of Māori participants' experiences would pass to a Māori researcher.^22^ Dissemination of study findings to participants was via a summary feedback video, which was preferred by participants over a written report or feedback hui (meeting). Ethical approval for the study was obtained from the Central Health and Disability Ethics Committee (New Zealand) and all study participants gave written consent.

## Results

This study's findings aligned with Camara Jones' framework for understanding racism and its impact on health service engagement.^12^ The core themes identified in the results reinforce this framework and have been grouped accordingly: Institutionalised Racism; Interpersonal Racism; and Internalised Racism (Table 2). The extent of programme engagement for Māori participants appeared to be related to the experiences of and responses to racism. This study also identified what was considered culturally appropriate care for participants.

**Table 2:** What affects engagement for Māori families?†

Theme	Example participant quotation
Institutionalised racism: substantial structural barriers and socio‐economic deprivation	And that's what I said at the family group conference – ‘I disagree with that because they were being fed’. It might not be healthy to some people. But at least they were eating
	I mean, she doesn't look like no Islander, she looks like a white girl. She's so fair
Interpersonal racism: cumulative effect of weight stigma and racism	…so firstly what I think happens for families who are vulnerable, and we sit in that category because we have disability in our family and I'm a single parent, and you layer that and all sorts of things that go with that and especially with [SON 1] because he's Māori and he's carrying weight so you add that on top of that… so there's a big huge vulnerability that sits in around that…
	… [my husband is] dark, he dresses like the rest of the [suburb] boys, which doesn't always come across to professionals as… they assume things that aren't necessarily true, but they put him in that basket a lot, into the sense that at times he wouldn't be able to pick up our own son's medication from the chemist. Yeah, and that used to piss me off. It was like “what?” But, yeah they just put you in that category. And it's, yeah, not fair
Internalised racism and biological determinism: perception that ancestry determines outcome	[referring to Māori ancestry] That's why he's built like he is
	… he was a bigger boy and I mean, you know, I'm big, his father's bigger than me so it's just in his [blood]

^†^ Data are from interviews with Māori whānau (families).

### Institutionalised racism: Substantial structural barriers and socio‐economic deprivation

Institutionalised racism was evident through the wide range of adverse events affecting many Māori participants and their families, which affected their capacity to engage with the service and the wider health system. Māori participants and their families frequently reported both acute and chronic life stressors and difficult socio‐economic conditions (Table 2).

*We didn't have a vehicle. And we were going to car‐pool with my aunty who had to take her son as well. And, um, she didn't have enough for all of us, you know. I just felt shit that I couldn't take him […] Unless I hitch hike with all of them*.

Systemic racism was demonstrated through the range of experiences evident within single families. For example, Māori or Pacific participants who were socially assigned by other people as NZ European experienced racism differently in the health system and wider society. Participants acknowledged that they were accorded societal advantage or disadvantage depending on how they were socially assigned by others.

*Um, when the two boys were going to school, cause one was dark and one was white, they used to get teased quite a bit because they didn't think they were brothers and that's really upsetting because why should they be different because one's dark and one's white, you know, because [SON 2] actually identifies with his culture just as much as [SON 1] does, and it's almost like he's worked twice as hard whereas [SON 1]'s dark and he doesn't care, you know […] I know a lot of people who name their children, um European names so they get ahead, and they are still do that today and it's really upsetting if that happens and disappointing*.

### Interpersonal racism: Cumulative effect of weight stigma and racism

While weight stigma was experienced by both Māori and non‐Māori participants, Māori participants reported compounding effects of weight and race stigma within their interactions with health professionals and felt targeted because of their weight and being Māori (Table 2). When asked about discrimination, one participant emotionally recounted her reaction to being negatively stereotyped by a health professional:

*I'm like ‘mmm, that makes you not want to go back to you… [tearful] it's probably true […] She's trying to do her job, but it's just how she said it[…] Yeah. I can still see her face now. I didn't like her. We didn't like her*.

Much of the interpersonal racism experienced by participants both within and outside the health system was centred around implicit bias and stereotyping, as a result of systemic racism. Participants were sometimes reluctant to articulate this as racism and instead described experiences of being judged or treated unfairly in the community (Table 2).

*… he said “Mum, I'm the only brown boy at school.” I said “no you're not. I'm sure there's another one.” […] he said “oh no, [classmate] is brown too, that's right,” and I said “see, you're not the only one,” but there's some kids there, white kids, who, I'm just assuming are a little bit racial. That might be because of the way their family, you know, obviously it's how their parents kind of probably are, and I know that their parents possibly don't have brown associations*.

However, participants also identified more explicit instances of racism occurring within and outside the health system (Table 2), resulting in suspicion of a range of government‐provided services in addition to the health system (Table 3).

**Table 3:** Participant responses to experiences of racism in terms of engagement with health services†

Participant response	Example participant quotations
Distrust of health services	I don't trust the health care system. Definitely don't. I record everything, I investigate everything, make sure that I'm happy with everything, and yeah
Renewed determination to engage with health services	I know there's people around that may, you know, everyone is judgemental, and they may be stereotyping or whatever, if they saw us maybe behave… I just think let people do that if they want, that's their shallow lives, they have no idea what we're about and it's not going to worry us what other people think
Disengagement with health services	I probably did need the referral, but because, part of it because I didn't know what they did or who they were. I wasn't keen on an outsider coming in, if you know what I mean. Um, an outside entity coming in. Like, um yeah and it had nothing to do with who was in it or anything like that. It was just yeah my kid needed some tough love

† +/−Other government services.

*Cops [police] may judge us, but they won't even know that they're judging us. Like, really in their intentions or hidden agendas, they judge us because of what I look like. They're driving, but really, they're going to pull you over. ‘Oh, we're just doing a random check’. I've done it, sort of driven past cops with this [hat and hooded jumper] on and then when you drive back past them with no hat and they don't even look at us. Yeah, my mate… I was driving with him and he, he saw a cop coming and he goes ‘bro, take your hat off, take your hat off’ and I go ‘aye, why is that’? ‘Coz some cops are coming, take it off’, and he took it off and then it made a difference – cop didn't look at us*.

### Internalised racism and biological determinism: Perception that ancestry determines outcome

Many participants with Māori children self‐attributed overweight and obesity to family genetics, or more broadly to their ancestry (Table 2). There was a perception that Māori and Pacific peoples are ‘naturally big’ but that this was not necessarily a concern. This perception of children being ‘born big’ or ‘solidly built’ was evident across both Māori and non‐Māori families, but it was specifically linked to ethnicity by Māori participants and parents of Māori children.

*I'm from, um my mother was a [family name] and they're a big family – she's 1 of 18 – and so, I mean if you know any of the [family name]’s, some of them are built like big brick shit houses, so we're used to it*.

These internalised ideas shaped attitudes about genetic propensity towards overweight and obesity, the perceived potential effectiveness of healthy lifestyle change, and therefore the perceived value of engaging with healthy lifestyle services.

Additionally, the internalisation of racist stereotypes highlights the relationship between external racism and internalised identity (Table 2).

*…There are a lot of us that, yeah, there are a lot of bad people that are brown and that too so the good ones that are brown will actually have it all too because that's what happens I suppose. But we do get judged, but I don't let that beat us though […]*

Although not directly linked to health system use, the experiences of these participants are the result of the ongoing effects of both believing and challenging internalised racism, which often led to a distrust of and disengagement with services (Table 3).

*I think that's why I, in the end, I don't know if I could trust them [hospital service] with any help so I don't go to them anymore*.

### What is ‘appropriate’ care?

Despite the past negative incidents of care many participants had experienced, participants were clear about what constituted ‘good care’. Care that was respectful, compassionate and dignified appeared to overcome some of the past negative experiences. Many participants reported receiving this type of care in Whānau Pakari, as well as in other areas of health care. When asked directly, participants reported that Whānau Pakari was culturally appropriate, and responses tended to be centred around the positive relationships developed with the delivery team rather than any tangible aspect of the service itself.

*Do you mean culturally? Yeah of course or I wouldn't have been… we would have opted out otherwise. Yeah, we wouldn't have been going, we'd have been making up all these excuses, well I know I would. You would have been ‘hey, oh just don't worry about going today, I'll say something’, that sort of thing. Yeah, nah, I did, I really enjoyed it. Like I say, the staff, they were awesome… Our experience with you guys was awesome, and with everyone else. Those are pretty straight up answers*.

Participants also spoke of respectful care they had received elsewhere in the health system. Likewise, it centred around the compassion and kindness of health‐care professionals, irrespective of their ethnicity.

*Yeah, no, the Māori lady… get another Māori to approach another Māori aye… yeah she kind of became someone that we could turn to if we needed anything, which was awesome, but she'd also throw some of it back in our face as well, where it got to the point where I was ‘I don't think I need you actually’. Aye, I think I might go to this side of the fence where I've got an awesome um white lady who is prepared to sit there, listen and help, you know, help me and my daughter big time*.

This data demonstrates positive experiences with relationship‐building in the health system and are examples of how respect and compassion are central to appropriate care.

## Discussion

This study found that the engagement of Māori families in Whānau Pakari was influenced by the effects of institutionalised racism, manifesting as socio‐economic deprivation and other differential barriers to access, and interpersonal and internalised racism and stigma experienced throughout the health system and society. Responses to these experiences included disengagement from Whānau Pakari, regardless of where the experience occurred. However, respectful and culturally appropriate care with an emphasis on positive relationship‐building may be a way to partially mitigate and resist past experiences of racism and weight stigma. Our data suggest that at the system and service level, health care that practices *manaakitanga* (the process of showing respect, support, and care for others) and *aroha* (love, compassion, empathy, kindness) as guiding principles can promote engagement. However, this is not always sufficient to retain families who are dealing with multiple complex challenges while also being faced with many barriers to making healthy lifestyle changes.^24^ Previous research has also suggested that there are multiple factors hindering engagement *external* to the service provision;^21^ this study reinforces the need to address wider social issues to enhance engagement and retention in services such as Whānau Pakari.

This study's findings are consistent with the limited previous literature on barriers to engagement in lifestyle interventions for Indigenous and other marginalised ethnic groups, as well as literature on health care access more generally which commonly identifies socio‐economic factors and racial discrimination as key barriers for Indigenous peoples.^8^, ^25^ The high rates of deprivation for Māori as a result of institutionalised racism are likely to contribute towards differential access to health services such as Whānau Pakari between Māori and NZ Europeans. While ‘personal circumstances’ are frequently identified as factors influencing attendance in multiple studies,^4^ a study of participant retention in a family‐led weight‐management programme for Pacific children with obesity specifically identified unpredictable life events such as deaths, illnesses and employment changes as key barriers which affected participation.^7^ Although some of these stressors are unpredictable, many are the result of household deprivation in participant communities. For example, it is difficult to address weight issues when food insecurity is a bigger threat to health and wellbeing due to the social conditions in which children and their families live. In NZ, almost one in five children live in households that are experiencing moderate–severe food insecurity.^26^ These factors are external to a healthy lifestyle service and were consistently identified as determinants of disengagement as families' priorities are forcibly and understandably changed.

While it was not identified as a factor affecting engagement in a systematic review of barriers and facilitators of engagement and retention in childhood obesity interventions,^4^ our study demonstrates that previous occurrences of racism have an enduring ability to influence seemingly unrelated interactions within the health system. Racism in NZ has previously been identified as a key determinant of health due to the ongoing effects of colonisation, contributing towards health loss and inequities between Māori and NZ Europeans^9^, ^15^ with a 2018 study demonstrating a dose–response relationship between the number of forms of discrimination experienced and negative health impacts.^15^ Our study suggests that for some Māori participants, various experiences of racism and weight stigma – regardless of where they occurred – affected subsequent engagement with the health system. Practically, this has important implications for clinical care – if participants have had stigmatising or discriminatory experiences elsewhere, this may affect engagement with other unrelated services.

Indigenous‐led services are critical in order to mitigate inequities.^27^ Ensuring that prevailing or ‘mainstream’ health‐care services are culturally appropriate and reflect the communities they serve is also important, in order to complement Indigenous‐led service provision. Previous literature on engagement in healthy life‐style programmes has cited a lack of ‘cultural appropriateness’ as a reason for lower engagement, without further elaboration.^4^ In our study, the most important facilitators of engagement for Indigenous groups were not concrete components in a ‘check‐list’ of appropriateness or competency.^25^, ^28^ Critically, the acceptability of Whānau Pakari for all participants, but particularly for Māori, stemmed from positive, respectful, trusting relationships between the programme deliverers and programme participants, as defined by the participants themselves.^21^ When participants chose to engage with Whānau Pakari despite historical experiences of weight stigma and racism, they cited the relationships developed with programme deliverers as the reason this engagement was successful. Previous studies have identified relationships and social connectedness as essential for acceptable services and enabling engagement with Indigenous groups,^25^, ^29^, ^30^, ^31^ especially in healthy lifestyle programmes.^4^ This is aligned with the strengths‐based concept of *cultural safety*, which refers to health care delivery with a focus on critical analysis of and reflection on the power imbalances in health care interactions between Indigenous patients and non‐Indigenous health care providers.^27^, ^28^, ^32^ A context of cultural safety can facilitate appropriate care.^27^ Past evaluations of the service have suggested that Whānau Pakari is considered culturally acceptable.^33^ The programme emphasis on general health and wellbeing rather than obesity is also likely to contribute towards the acceptability of the programme for Māori.^29^

A strength of this study is the strong representation from Māori participants and those who chose not to engage with the service at all. Our high recruitment rate was due to a concerted effort to overcome common barriers to research participation; this included text reminders, home‐based interviews, a mixed Māori‐NZ European interview and research team, establishing rapport with participants before commencing, and a *koha* (gift) which recognised the time and effort given by participants as a sign of reciprocity. The research process was designed to be a positive, respectful experience for participants. The analysis was undertaken with Māori and non‐Māori researchers who had agreed parameters for respectful contributions.^22^

The research may not be generalisable to other groups in NZ and globally to other Indigenous populations; however, it does identify a number of issues that have commonalities in Indigenous peoples' experiences globally. A limitation is the ability of this study to capture the heterogeneity of Māori experience. Half of the interviews were with participants who had Māori children involved in Whānau Pakari, and this also included interviews with NZ European parents and caregivers of Māori children. While this perhaps influenced how participants perceived their experiences, it also reflects the lived realities of Māori children growing up in contemporary NZ. Finally, the majority of participants were mothers or female caregivers, which may have affected the results. For the small number of male parents who participated, answers did not appear to differ from female parents' answers.

## Conclusions

In conclusion, experiences of racism at institutionalised, interpersonal and internalised levels affects engagement for Māori families engaged in a healthy lifestyle programme. Past negative experiences elsewhere in the health care system affected engagement with the service. While participation in the programme was identified as a positive experience for most participants, this was insufficient to achieve sustained engagement for some families due to external competing priorities. Racism that occurs elsewhere in the health system or in wider society may have ongoing effects with regards to subsequent engagement with other health services, despite their acceptability, and until these issues are addressed, inequities in service engagement between Indigenous and non‐Indigenous groups are likely to persist. While it may not be enough to address the effects of racial discrimination more generally, compassionate, respectful, culturally safe care focusing on positive relationship‐building can help mitigate some of the impact of racism on engagement in prevailing health‐care services.
